# Effects of a dietary isoquinoline alkaloids blend on gut antioxidant capacity and gut barrier of young broilers

**DOI:** 10.1016/j.psj.2024.103654

**Published:** 2024-03-14

**Authors:** Vasileios Paraskeuas, Anja Pastor, Tobias Steiner, Konstantinos C. Mountzouris

**Affiliations:** ⁎ Laboratory of Nutritional Physiology and Feeding, Department of Animal Science, School of Animal Biosciences, Agricultural University of Athens, Athens 11855, Greece; † Phytobiotics Futterzusatzstoffe GmbH, Eltville, Germany

**Keywords:** isoquinoline alkaloid, phytogenic, young broiler, gut cytoprotection, gut barrier

## Abstract

Extensive mechanistic evidence to support the beneficial function of dietary phytobiotic applications for broiler performance, gut function and health is highly warranted. In particular, for isoquinoline alkaloids (**IQ**) the underlying mechanisms related to critical gut homeostasis components such as cytoprotection and gut barrier are scarce, especially for young broilers at the starter growth stage (d1–10). The aim of this study was to investigate the effect of a standardized blend of IQs on the relative gene expression of critical biomarkers relevant for antioxidant response and barrier function along the intestine of young broilers at the end of starter growth phase. For this purpose, 182 one-day-old Ross 308 broilers were allocated in 2 treatments with 7 replicates of 13 broilers each: control diet-no other additions (**NC**), and control diet containing a standardized blend of IQs at 200 mg/kg of diet (M) for the starter growth period (1–10d). The results revealed that the IQs blend significantly upregulated (*P <* 0.05) the expression of genes related to antioxidant response in all intestinal segments. Moreover, the IQs blend enhanced (*P <* 0.05) gut barrier components primarily at duodenal level. In conclusion, the blend of IQs beneficially affected critical pathway components relevant for the gut antioxidant capacity and barrier along the intestine of young broilers.

## INTRODUCTION

The successful overall management of highly productive animals such as the broiler chickens is a prerequisite requirement for sustainable livestock production. During the starter period (1–10d), broilers are in a sensitive physiological state, characterized by rapidly developing organs, digestive immaturity and an undeveloped immune function (Del Vesco et al., 2017). At the starter growth phase, the broiler's high growth potential coincides with increased risks of exposure to various exterior stressors and appearance of disturbances in their gut function (Panda and Cherian. 2014; Lauridsen et al., 2019).

Phytobiotics are compounds that originate mostly from herbs and spices. The majority of such compounds are currently categorized as flavoring feed additives according to the current European Union regulation [Regulation (EC) No 1831/2003]. Isoquinoline alkaloids (**IQs**), including quaternary-benzo(c)phenanthridine alkaloids (e.g., chelerythrine and sanguinarine) and protopine alkaloids (e.g., protopine and allocryptopine) sourced from plant materials, for example, *Macleaya cordata*, have been reported to exhibit growth promoting effects in broilers (Khadem et al., 2014; Xue et al., 2017; Kikusato et al., 2021b; Khongthong et al., 2023) that may be attributed to their ability to improve intestinal barrier function (Dong et al., 2020; Ibrahim et al., 2020; Liu et al., 2022; Khongthong et al., 2023; Song et al., 2023), systemic (Liu et al., 2022) and intestinal inflammation (Khadem et al., 2014; Kikusato et al., 2021b; Khongthong et al., 2023; Song et al., 2023), gut microbiota composition (Wang et al., 2022; Song et al., 2023) and antioxidant capacity in blood and liver (Kikusato et al., 2021b; Liu et al., 2022).

However, even though in recent years the scientific interest about phytobiotic compounds rich in IQs in broiler nutrition has been growing, the available literature data on their functional roles for gut function and health along the intestine are far from being conclusive.

Recent research evidence suggests that phytobiotics can enhance broiler gut barrier components via promoting the expression of tight junction (**TJ**) proteins (e.g., occludin, claudins, zonula occludens) which regulate the permeability and seal the paracellular space between the enterocytes of the intestinal barrier (Du et al., 2016; Zou et al., 2016; Paraskeuas and Mountzouris, 2019a; Paraskeuas and Mountzouris, 2019b; Liu et al., 2022; Pham et al., 2022; Khongthong et al., 2023; Liu et al., 2023; Song et al., 2023). Moreover, phytobiotics may activate the nuclear factor (erythroid-derived 2)-like 2 (**Nrf2**) pathway which is a key regulator of cellular antioxidant response that triggers the expression of antioxidant enzymes in broiler intestinal enterocytes (Mueller et al., 2012; Griela et al., 2021; Liu et al., 2022; Griela and Mountzouris 2023; Liu et al., 2023). In particular, Nrf2 is a transcription factor responsible for the regulation of antioxidant response. Potential inducers such as phytobiotics, trigger its translocation from cytoplasm to the nucleus and lead to the transcription of cytoprotective genes (Mountzouris et al. 2020).

Given the above, it is clear that IQs merit further investigation for their effects on the gut antioxidant response and barrier function along the intestine of young broilers. Therefore, the aim of the present study was to investigate the dietary effect of a standardized IQs blend on broiler growth performance and the relative expression of genes related to Nrf2 pathway and tight junction proteins along the gut of broilers during their starter growth phase (1–10d).

## MATERIALS AND METHODS

### Animal and diets

Housing, management and care of the animals complied with the current European Union Directive on the protection of animals used for scientific purposes (EC 43/2007, EU 63/ 2010; Council of the European Union 2007, 2010), and the experimental protocol was approved (No7/03032020) by the Bioethics Committee of the Agricultural University of Athens (**AUA**), Greece. One hundred eighty-two male 1-day-old Ross 308 broilers were vaccinated for Marek's disease, infectious bronchitis and Newcastle disease. Broilers were obtained from a commercial hatchery and randomly allocated in 2 experimental treatments with 7 replicates per treatment. Each replicate had 13 broilers. All experimental treatments received a maize-soyabean meal basal diet in mash form, formulated according to Ross 308 nutrient requirements for the starter growth phase (d1–10) with coccidiostat included. The ingredients and the chemical composition of the experimental diet are presented in Table 1. Broilers were allocated to 2 treatments: **NC** (basal diet – no other additions), and **M** (basal diet containing 200 ppm of a standardized blend of isoquinoline alkaloids, provided as Sangrovit Feed (Phytobiotics Futterzusatzstoffe GmbH, Germany). Each treatment replicate was assigned to a clean floor pen (2 m^2^), and birds were raised on rice-hull litter. Birds had 24 h light during d 1, then 23 h light and 1 h dark until d 7, and from d 8 to 10 the lighting program was set to 18 h light and 6 h dark. Room temperature was in accordance with the Management Guide recommendations for Ross 308 broilers. Throughout the experiment diets and water were available ad libitum.

**Table 1:** Ingredient (g/kg) and calculated chemical composition (g/kg as fed) of the basal experimental diets.

Ingredients	Starter (d 1–10)
Maize	522.2
Soybean meal (44%)	349.1
Soy protein concentrate^1^	50.0
Soy oil	34.6
Limestone	12.7
Mono calcium phosphate	15.0
Salt (NaCl)	2.1
Sodium bicarbonate	2.2
L-lysine-HCL	2.7
DL-methionine	3.8
L-threonine	1.1
Vitamin premix^2^	2.0
Mineral premix^3^	2.0
Coccidiostat^4^	0.5
Isoquinoline alkaloids blend^5^	-
Calculated chemical composition	
AME_n_,(MJ/kg diet)	12.6
Dry matter (%)	900.1
Crude protein (%)	230.0
Ether extract (%)	58.2
Crude fiber (%)	37.6
Lysine (g/kg)	14.4
TSAA (methionine + cysteine) (g/kg)	10.8
Threonine (g/kg)	9.7
Calcium (g/kg)	9.6
Available phosphorus (g/kg)	4.8
Sodium (g/kg)	1.6

1 Soy Protein concentrate with 530 g crude protein/kg (Alpha Soy 530, Agilia Europe, Skjernvej 42, DK-6920, Videbaek, Denmark).

2 The vitamin premix for the starter period (Rovimix 11 Bro Basic, DSM, Netherlands) provided per kg of diet: 3.6 mg retinol (Vit.A), 100 μg cholecalciferol (Vit.D_3_), 80 mg Vit.E, 9 mg Menadione (Vit.K_3_), 3 mg Thiamine,7 mg Riboflavin, 6 mg Pyridoxine, 25 μg Cyanocobalamin, 50 mg Nicotinic acid, 15 mg Pantothenic acid, 1.5 mg Folic acid, 150 μg Biotin. The vitamin premix for the grower and finisher period (Rovimix 12 Bro Basic, DSM, Netherlands) provided per kg of diet: 3.6 mg retinol (Vit.A), 75 μg cholecalciferol (Vit.D_3_), 50 mg Vit.E, 7 mg Menadione (Vit.K_3_), 3 mg Thiamine, 6 mg Riboflavin, 6 mg Pyridoxine, 25 μg Cyanocobalamin, 40 mg Nicotinic acid, 12 mg Pantothenic acid, 1.2 mg Folic acid, 150 μg Biotin.

3 The mineral (Rovimix Bro M, DSM, Netherlands) provided per kg of diet: 400 mg choline chloride, 250 μg Co, 1.5 mg I, 300 μg Se, 50 mg Fe, 130 mg Mn, 20 mg Cu and 100 mg Zn.

4 Maxiban G160, Elanco, Elli Lilly and Company, Clinton Laboratories, Clinton, Indiana, USA.

5 Standardized blend of isoquinoline alkaloids, provided as Sangrovit Feed, Phytobiotics Futterzusatzstoffe GmbH, Germany (treatment M).

### Growth Performance Responses

Body weight **(BW**) and feed intake (**FI**) were evaluated for the first ten days of the experiment (1–10d). Mortality was recorded daily. In addition, body weight gain (**BWG**) and feed conversion ratio (**FCR**) were also calculated for the starter growth period.

### RNA Isolation and Reverse-Transcription PCR

At 10 d, 1 broiler per pen was euthanized, the intestinal segments (duodenum, jejunum, ileum, ceca) were sampled, longitudinally opened and the luminal digesta were removed. Afterwards, the segments were washed thoroughly in 10 mL ice cold PBS-EDTA (10 mM) solution (pH = 7.2) and a small piece (about 70–100 mg) was cut off and placed in sterile eppendorf tubes. Eventually, the total RNA from the intestinal segments was extracted using the NucleoZOL Reagent (Macherey-Nagel GmbH; Co. KG, Germany), according to the manufacturer's protocol. RNA quantity and quality was determined by spectrophotometry (NanoDrop-1,000, Thermo Fisher Scientific, Waltham, United Kingdom).

Treatment with DNAse ensured the removal from the RNA samples of any contaminating genomic DNA. Ten μg of RNA were resuspended with 1 unit of DNase I (M0303, New England Biolabs Inc, Ipswich, UK) and 10 μL of 10x DNAse buffer to a final volume of 100 µL with the addition of DEPC water, for 20 minutes at 37°C. Before the DNAse inactivation at 75°C for 10 min, EDTA was added to a final concentration of 5 mM to protect RNA from being degraded during enzyme inactivation. RNA integrity was checked by agarose gel electrophoresis.

For cDNA preparation, 500 ng of total RNA from each sample were reverse transcribed to cDNA by PrimeScript RT Reagent Kit (Perfect Real Time, Takara Bio Inc., Shiga-Ken, Japan) according to the manufacturer's recommendations. All cDNAs were then stored at –20°C.

The *Gallus gallus* genes below were investigated: Glyceraldehyde 3-phosphate dehydrogenase (***GAPDH***), actin beta (***ACTB***), nuclear factor (erythroid-derived 2)-like 2 (***Nrf2***), kelch like ECH associated protein 1 (***Keap1***), catalase (***CAT***), superoxide dismutase 1 (***SOD1***), glutathione peroxidase 2 (***GPX2***), glutathione peroxidase 7 (***GPX7***), glutathione S-transferase-α (***GST)***, glutathione reductase (***GSR***), NAD(P)H quinone dehydrogenase 1 (***NQO1***), heme oxygenase 1 (***HMOX***1), peroxiredoxin 1 (***PRDX1***), zonula occludens-1 (***ZO1***), zonula occludens-2 (***ZO2***), \claudin-1 (***CLDN1***), claudin-2 (***CLDN2***), claudin-5 (***CLDN5***), occluding (***OCLN***) and mucin-2 (***MUC2***), by quantitative real-time PCR Suitable primers were designed using the GenBank sequences deposited on the National Center for Biotechnology Information and US National Library of Medicine (**NCBI**) shown in Table 2. Primers were checked using the PRIMER BLAST algorithm for *Gallus gallus* mRNA databases to ensure that there was a unique amplicon.

**Table 2:** Oligonucleotide primers used for gene expression of selected targets by quantitative real time PCR.

Target^1^	Primer sequence (5′-3′)^2^	Annealing temperature (⁰C)	PCR product size (bp)	GenBank (NCBI reference sequence)
GAPDH	F: ACTTTGGCATTGTGGAGGGT R: GGACGCTGGGATGATGTTCT	59.5	131	NM_204305.1
ACTB	F: CACAGATCATGTTTGAGACCTT R: CATCACAATACCAGTGGTACG	60	101	NM_205518.1
Nrf2 pathway related genes and heat-shock proteins				
NRF2	F: AGACGCTTTCTTCAGGGGTAG R: AAAAACTTCACGCCTTGCCC	60	285	NM_205117.1
KEAP1	F: GGTTACGATGGGACGGATCA R: CACGTAGATCTTGCCCTGGT	62	135	XM_025145847.1
CAT	F: ACCAAGTACTGCAAGGCGAA R: TGAGGGTTCCTCTTCTGGCT	60	245	NM_001031215
SOD1	F: AGGGGGTCATCCACTTCC R: CCCATTTGTGTTGTCTCCAA	60	122	NM_205064.1
GPX2	F: GAGCCCAACTTCACCCTGTT R: CTTCAGGTAGGCGAAGACGG	62	75	NM_001277854.1
GPX7	F: GGCTCGGTGTCGTTAGTTGT R: GCCCAAACTGATTGCATGGG	60	139	NM_001163245.1
GST	F: GCCTGACTTCAGTCCTTGGT R: CCACCGAATTGACTCCATCT	60	138	NM_001001776.1
GSR	F: GTGGATCCCCACAACCATGT R: CAGACATCACCGATGGCGTA	62	80	XM_015276627.1
NQO1	F: GAGCGAAGTTCAGCCCAGT R: ATGGCGTGGTTGAAAGAGGT	60.5	150	NM_001277619.1
HMOX1	F: ACACCCGCTATTTGGGAGAC R: GAACTTGGTGGCGTTGGAGA	62	134	NM_205344.1
PRDX1	F: CTGCTGGAGTGCGGATTGT R: GCTGTGGCAGTAAAATCAGGG	61	105	NM_001271932.1
Gut barrier integrity genes				
ZO1	F: CTTCAGGTGTTTCTCTTCCTCCTC R: CTGTGGTTTCATGGCTGGATC	59.5	131	XM_413773
ZO2	F: CGGCAGCTATCAGACCACTC R: CACAGACCAGCAAGCCTACAG	59.5	87	NM_204918
CLDN1	F: CTGATTGCTTCCAACCAG R: CAGGTCAAACAGAGGTACAAG	59.5	140	NM_001013611
CLDN2	F: CAAGGACCGAGTGGCAGTG R: TTTGATGGAGGGCTGAGGA	62	289	NM_001277622.1
CLDN5	F: CATCACTTCTCCTTCGTCAGC R: GCACAAAGATCTCCCAGGTC	59.5	111	NM_204201
OCLN	F: TCATCGCCTCCATCGTCTAC R: TCTTACTGCGCGTCTTCTGG	62	240	NM_205128.1
MUC2	F: GCTGATTGTCACTCACGCCTT R: ATCTGCCTGAATCACAGGTGC	62	442	XM_015274015.1

1 GAPDH: glyceraldehyde 3-phosphate dehydrogenase; ACTB: actin, beta; Nrf2: nuclear factor (erythroid-derived 2)-like 2; Keap1: kelch like ECH associated protein 1; CAT: catalase; SOD1 = superoxide dismutase 1; GPX2 = glutathione peroxidase 2; GPX7 = glutathione peroxidase 7; GST**=** Glutathione S-transferase-α**;**; GSR = Glutathione reductase; NQO1 = NAD(P)H quinone dehydrogenase 1; HMOX1 = heme oxygenase 1; PRDX1 = Peroxiredoxin 1; ZO1=zonula occludens-1; ZO2 = zonula occludens-2; CLDN1 = claudin-1; CLDN2= claudin-2; CLDN5 = claudin-5; OCLN = occludin; MUC2= mucin-2

2 F: forward; R: reverse.

Real-time PCR was performed in 96 well microplates with a SaCycler-96 Real-Time PCR System (Sacace Biotechnologies s.r.l.) and FastGene IC Green 2 × qPCR universal mix (Nippon Genetics, Tokyo, Japan). Each reaction contained 12.5 ng RNA equivalents as well as 200 nM of forward and reverse primers for each gene.

The reactions were incubated at 50°C for 2 min, 95°C for 2 min, followed by 40 cycles of 95°C for 15s 59,5 to 64.5°C (depends on the target gene) for 15 s, 72°C for 1 min. This was followed by a melt curve analysis to determine the reaction specificity. Each sample was measured in duplicates. Relative expression ratios were calculated according to Pfaffl. (2001) and were adapted for the multi-reference genes normalization procedure according to Hellemans et al., (2007) using *GAPDH* and *ACTB* as reference genes.

### Statistical Analysis

Growth performance responses were analyzed on a pen basis and relative gene expression data evaluation was based on individual broilers. Data were analyzed with *t* test procedure. Statistical significance was determined at *P* < 0.05. All statistical analyses were done using the SPSS for Windows Statistical Package Program (SPSS Inc., Chicago, IL).

## RESULTS

### Growth Performance Responses

The broiler growth performance responses during the starter period (d 1–10) are shown in Table 3. There were no significant (*P >* 0.05) differences in BW, BWG, FI, and FCR between the 2 experimental treatments.

**Table 3:** Broiler growth performance responses during starter growth period (1–10d).

	Experimental treatments^1^			
Components^2^	NC	M	SEM^3^	*P*-value^4^
Starter (d 1–10)				
BW^5^ (g)	243.4	247.7	2.73	0.452
BWG (g)	195.2	199.2	2.68	0.478
FI (g)	251.3	261.9	6.75	0.452
FCR (g) Mortality (%)^6^	1.29 1.19	1.32 0.00	0.033 -	0.685 -

1 NC (basal diet - no other additives) and M (basal diet + 200 ppm of a standardized blend of isoquinoline alkaloids, provided as Sangrovit Feed, Phytobiotics Futterzusatzstoffe GmbH, Germany).

2 Data represent treatment means from n=7 replicate floor pens per treatment.

3 Pooled standard error of means.

4 The statistical analysis tests the differences between treatments with the ANOVA-Compare of means procedure using the SPSS for Windows statistical package. Means within the same row with different superscripts (a, b) differ significantly (*P<* 0.05).

5 BW: body weight; BWG: body weight gain; FI: feed intake; FCR: feed conversion ratio.

6 The mortality rate was very low and it cannot be statically evaluated.

*Profile of the relative expression of Nrf2 pathway and gut barrier related genes along the broiler intestine.*

### Duodenum

In the duodenum, the relative expression of genes related to the Nrf2 pathway (*Keap1, CAT, SOD, GPX7, GSR, NQO1, HMOX1, PRDX1*) and gut barrier integrity (*ZO1, ZO2, CLDN5, OCLN, MUC2*), were significantly different between the 2 treatments (Table 4). The inclusion of IQs blend in broiler diets (M), increased the relative expression of *Keap1* (*P =* 0.001), *CAT* (*P =* 0.006), *SOD* (*P =* 0.005), *GPX7* (*P <* 0.001), *GSR* (*P =* 0.010) *NQO1* (*P* = 0.002), *HMOX1* (*P* = 0.010) and *PRDX1* (*P = 0.044*) compared to treatment NC. Moreover, the expression levels of *ZO1* (*P =* 0.002), *ZO2* (*P =* 0.002), *CLDN5* (*P =* 0.021), *OCLN* (*P =* 0.020) and *MUC2* (*P =* 0.001), were higher in treatment M compared to NC (Table 4).

**Table 4:** Relative gene expression of gut barrier integrity and Nrf2 pathway related genes in duodenum of 10d old broilers.

Genes^1^	Treatments^2^		Statistics	
	NC	M	SEM^3^	*p-value*^4^
10d				
Duodenum				
Nrf2 pathway				
Nrf2	0.96	1.94	0.282	0.102
Keap1	0.56^Β^	1.86^Α^	0.214	0.001
CAT	0.73^Β^	1.40^Α^	0.133	0.006
SOD	0.70^B^	1.88^A^	0.232	0.005
GPX2	0.75	2.17	0.468	0.134
GPX7	0.69^B^	1.5^A^	0.131	<0.001
GST	1.12	1.35	0.121	0.358
GSR	0.88^b^	1.45^a^	0.116	0.014
NQO1	0.68^B^	1.14^A^	0.078	0.002
HMOX1	0.79^B^	1.26^A^	0.099	0.010
PRDX1	0.83^b^	1.22^a^	0.100	0.044
Gut barrier integrity				
ZO1	0.71^B^	1.41^A^	0.126	0.002
ZO2	0.68^B^	1.51^A^	0.144	0.002
CLDN1	1.02	1.19	0.098	0.397
CLDN2	0.83	1.29	0.135	0.090
CLDN5	0.74^b^	1.36^a^	0.130	0.021
OCLN	0.73^b^	1.31^a^	0.124	0.020
MUC2	0.70^B^	2.07^A^	0.235	0.001

1 Relative expression ratios of target genes were calculated according to Pfaffl. (2001) adapted for the multi-reference genes normalization procedure according to Hellemans et al., (2007) using glyceraldehyde 3-phosphate dehydrogenase (*GAPDH*) and actin beta (*ACTB*) as reference genes.

2 NC (basal diet - no other additives) and M (basal diet + 200 ppm of a standardized blend of isoquinoline alkaloids, provided as Sangrovit Feed, Phytobiotics Futterzusatzstoffe GmbH, Germany).

3 Pooled standard error of means.

4 Data represent treatment means for 7 broilers per treatment. Means within the same row with different superscripts (a, b, c or A, B, C) within the same row differ significantly (*P <* 0.05 or 0.01).

### Jejunum

In the jejunum, the relative expression levels of *Nrf2* (*P =* 0.024), *Keap1* (*P =* 0.011), *GPX2* (*P =* 0.002), *GST* (*P =* 0.043) and *HMOX1* (*P <* 0.001) were increased in treatment M compared to NC treatment, respectively. The genes relevant for the gut barrier integrity did not (*P >* 0.05) differ between the 2 treatments (Table 5).

**Table 5:** Relative gene expression of gut barrier integrity and Nrf2 pathway related genes in jejunum of 10d old broilers.

Genes^1^	Treatments^2^		Statistics	
	NC	M	SEM^3^	*p-value*^4^
10d				
Jejunum				
Nrf2 pathway				
Nrf2	0.71^b^	1.48^a^	0.171	0.024
Keap1	0.53^b^	1.47^a^	0.188	0.011
CAT	0.90	1.15	0.101	0.230
SOD	0.63	1.40	0.222	0.082
GPX2	0.59^B^	1.60^A^	0.174	0.002
GPX7	0.80	1.21	0.140	0.147
GST	0.33^b^	1.71^A^	0.324	0.043
GSR	0.81	0.98	0.141	0.579
NQO1	0.86	1.12	0.132	0.327
HMOX1	0.32^B^	1.82^A^	0.255	<0.001
PRDX1	0.88	1.00	0.112	0.601
Gut barrier integrity				
ZO1	0.98	1.19	0.135	0.475
ZO2	0.90	1.32	0.167	0.233
CLDN1	1.08	1.17	0.126	0.735
CLDN2	0.95	1.24	0.165	0.392
CLDN5	0.95	1.25	0.169	0.398
OCLN	1.10	1.23	0.173	0.717
MUC2	0.93	1.06	0.105	0.563

1 Relative expression ratios of target genes were calculated according to Pfaffl. (2001) adapted for the multi-reference genes normalization procedure according to Hellemans et al., (2007) using glyceraldehyde 3-phosphate dehydrogenase (*GAPDH*) and actin beta (*ACTB*) as reference genes.

2 NC (basal diet - no other additives) and M (basal diet + 200 ppm of a standardized blend of isoquinoline alkaloids, provided as Sangrovit Feed, Phytobiotics Futterzusatzstoffe GmbH, Germany).

3 Pooled standard error of means.

4 Data represent treatment means for 7 broilers per treatment. Means within the same row with different superscripts (a, b, c or A, B, C) within the same row differ significantly (*P <* 0.05 or 0.01).

### Ileum

In the ileum, the dietary supplementation of the IQs blend increased the relative expression levels of *Keap1* (*P =* 0.029), *CAT* (*P =* 0.012), *SOD* (*P =* 0.025), *GPX2* (*P =* 0.001), *GSR* (*P <* 0.001), *NQO1* (*P =* 0.006) and *HMOX1* (*P =* 0.005), compared to treatment NC. Regarding the gut integrity genes studied, the expression levels of *CLDN1* (*P =* 0.029) and *CLDN5* (*P =* 0.049), were increased with the IQs inclusion compared to treatment NC (Table 6).

**Table 6:** Relative gene expression of gut barrier integrity and Nrf2 pathway related genes in ileum of 10d old broilers.

Genes^1^	Treatments^2^		Statistics	
	NC	M	SEM^3^	*p-value*^4^
10d				
Ileum				
Nrf2 pathway				
Nrf2	0.92	0.83	0.147	0.783
Keap1	0.80^b^	1.40^a^	0.133	0.029
CAT	0.63^b^	1.39^a^	0.166	0.014
SOD	0.56^b^	1.56^a^	0.218	0.025
GPX2	0.67^B^	1.66^A^	0.178	0.001
GPX7	0.62	1.00	0.128	0.152
GST	1.08	0.98	0.090	0.582
GSR	0.23^B^	2.35^A^	0.320	<0.001
NQO1	0.50^B^	1.74^A^	0.249	0.006
HMOX1	0.46^B^	1.70^A^	0.228	0.005
PRDX1	0.85	1.32	0.184	0.214
Gut barrier integrity				
ZO1	0.86	1.24	0.147	0.205
ZO2	0.79	1.54	0.201	0.075
CLDN1	0.76^b^	1.40^a^	0.142	0.029
CLDN2	0.57	1.09	0.143	0.068
CLDN5	0.65^b^	1.13^a^	0.127	0.049
OCLN	0.86	1.37	0.162	0.135
MUC2	1.08	1.15	0.113	0.777

1 Relative expression ratios of target genes were calculated according to Pfaffl. (2001) adapted for the multi-reference genes normalization procedure according to Hellemans et al., (2007) using glyceraldehyde 3-phosphate dehydrogenase (*GAPDH*) and actin beta (*ACTB*) as reference genes.

2 NC (basal diet - no other additives) and M (basal diet + 200 ppm of a standardized blend of isoquinoline alkaloids, provided as Sangrovit Feed, Phytobiotics Futterzusatzstoffe GmbH, Germany).

3 Pooled standard error of means.

4 Data represent treatment means for 7 broilers per treatment. Means within the same row with different superscripts (a, b, c or A, B, C) within the same row differ significantly (*P <* 0.05 or 0.01).

### Ceca

In the ceca, the relative expression levels of *CAT* (*P* = 0.012), *SOD* (*P* = 0.003), *GST* (*P* = 0.002), *GSR* (*P =* 0.003) and *PRDX1* (*P =* 0.007) were increased with IQs addition compared to treatment NC. Finally, there were no significant (*P >* 0.05) differences in the relative expression levels of the gut barrier genes studied, between the 2 experimental treatments (Table 7).

**Table 7:** Relative gene expression of gut barrier integrity and Nrf2 pathway related genes in ceca of 10d old broilers.

Genes^1^	Treatments^2^		Statistics	
	NC	M	SEM^3^	*p-value*^4^
10d				
Ceca				
Nrf2 pathway				
Nrf2	0.77	1.19	0.164	0.215
Keap1	1.08	1.05	0.102	0.884
CAT	0.74^b^	1.06^a^	0.069	0.012
SOD	0.50^B^	1.28^A^	0.140	0.003
GPX2	0.64	1.35	0.206	0.084
GPX7	1.15	0.99	0.072	0.293
GST	0.61^B^	1.11^A^	0.094	0.002
GSR	0.37^B^	2.15^A^	0.309	0.003
NQO1	1.06	0.93	0.123	0.620
HMOX1	0.86	1.70	0.319	0.200
PRDX1	0.52^B^	0.94^A^	0.086	0.007
Gut barrier integrity				
ZO1	0.97	0.93	0.086	0.801
ZO2	0.86	1.01	0.101	0.475
CLDN1	1.09	0.78	0.110	0.161
CLDN2	1.28	1.33	0.270	0.933
CLDN5	1.03	1.19	0.128	0.569
OCLN	0.81	0.99	0.139	0.556
MUC2	1.63	1.26	0.263	0.505

1 Relative expression ratios of target genes were calculated according to Pfaffl. (2001) adapted for the multi-reference genes normalization procedure according to Hellemans et al., (2007) using glyceraldehyde 3-phosphate dehydrogenase (*GAPDH*) and actin beta (*ACTB*) as reference genes.

2 NC (basal diet - no other additives) and M (basal diet + 200 ppm of a standardized blend of isoquinoline alkaloids, provided as Sangrovit Feed, Phytobiotics Futterzusatzstoffe GmbH, Germany).

3 Pooled standard error of means.

4 Data represent treatment means for 7 broilers per treatment. Means within the same row with different superscripts (a, b, c or A, B, C) within the same row differ significantly (*P <* 0.05 or 0.01).

## DISCUSSION

It is understood that especially during the sensitive starter growth phase of fast-growing broiler chickens, gut capacity to keep the levels of free radicals within the cells under control is of paramount importance to prevent against the detrimental consequences of oxidative stress (Panda and Cherian. 2014; Del Vesco et al., 2017; Lauridsen et al., 2019). Equally important for gut and overall broiler health is the maintenance of an effective gut barrier that facilitates the digestive-absorptive processes and at the same time controls the paracellular transport between the epithelial cells via tight junction components (Ulluwishewa et al 2011) to prevent undesirable conditions that could lead to leaky gut (Wan et al., 2019; Zhang et al., 2022).

The beneficial role of phytobiotic compounds for gut function and health is getting increasingly recognized (Du et al., 2016; Paraskeuas and Mountzouris 2019a; Pham et al., 2022). However, it is also understood that within the context of overall broiler diets, phytobiotic functions would be mostly related to their bioactive component composition as well as their inclusion level in the diet (Mountzouris et al., 2020; El-Hack et al., 2022).

Recent scientific literature supports the dietary supplementation of IQs derived from *Macleaya cordata* in broiler diets for the IQs positive effects on performance, blood antioxidant status, inflammatory response and intestinal microbiota composition mainly at the grower and finisher growth periods and at specific intestinal sites (Kikusato et al., 2021b; Wang et al., 2022; Khongthong et al., 2023; Song et al., 2023). Therefore, this work focused, beyond registering performance responses, to enhance our understanding on molecular events relevant for gut antioxidant capacity and gut barrier integrity, along the broiler gut, during the early life stage of broilers.

In this sense, critical genes belonging to the Nrf2 pathway and gut epithelial tight junction components were profiled along the gut of 10d-old broilers. The functions and the gene members investigated have been addressed in detail in our previous studies (Mountzouris et al., 2020; Brouklogiannis et al., 2023). In brief, with respect to gut antioxidant capacity the transcription factor Nrf2 along with its main pathway components (i.e., *Keap1* and the enzymes *CAT, SOD, GPX2, NQO1, GST, HMOX1, PRDX1, GSR, GPX7*) is known to be mainly responsible for the cellular inducible cyto-protection (Mountzouris et al 2020; Griela et al., 2021). On the other hand, gut barrier components such as mucin (***MUC2***) and tight junction components such as *ZO1, ZO2, CLDN1, CLDN2,* and *CLDN5* are mostly responsible for the epithelial barrier and the sealing of the paracellular space between adjacent epithelial cells (Suzuki and Hara, 2011; Song et al., 2014).

In this work it was shown that the supplementation of the IQs blend did not affect broiler growth performance at the starter phase compared to the control, overall mortality was low (0.67%) and no adverse effects were noted. Generally, phytobiotic benefits in performance have been evidenced after the starter phase and mostly in the finisher phase (Paraskeuas et al., 2016; Griela et al., 2021; Pham et al., 2022). In particular, phytobiotics based on *Macleaya cordata* extracts, have been found to increase BWG and improve FCR when added at 100 mg/kg (Khadem et al., 2014; Kikusato et al., 2021b; Khongtong et al., 2023), 150 mg/kg (Xue et al., 2017), 350 mg/kg (Song et al., 2023) and 600 mg/kg (Liu et al., 2022). In each of the above cases, the positive effects of the phytobiotics on broiler growth performance were observed in the grower and finisher periods and overall. On the other hand, in the study of Wang et al., (2022), the dietary *Macleaya cordata extract* supplementation in yellow-feathered broiler diets at 1,000 mg/kg did not improve FCR at all the experimental periods. Moreover, phytobiotics based on IQs were reported to improve body weight gain and feed conversion efficiency in young broilers due to increased intestinal villi height and gut absorption capacity (Vieira et al., 2008; Juskiewicz et al., 2011). Many factors including the actual composition and concentration of phytobiotic components, the different dietary inclusion levels, broiler genotypes and age and the differences in the experimental conditions could possibly explain the discrepancies noted between various studies.

With respect to the IQs blend from *Macleaya cordata* used in this work, sanguinarine and chelerythrine, have exerted growth promoting effects (Lee et al., 2015; Liu et al., 2020) when added in broiler diets due to their anti-inflammatory (Niu et al., 2012; Zhang et al., 2020) and antioxidant (Chatuvredi et al., 1997; Matkar et al., 2008) properties. However, the underlying mechanisms related to the metabolic pathways relevant for the immune and antioxidant response, which could possibly explain the beneficial effects of these compounds still remain unclear.

Phytobiotics are known to possess antioxidant properties by directly scavenging reactive oxygen species (**ROS**) or via the prevention of ROS formation via the up-regulation of gene expression levels of multiple Nrf2 pathway antioxidant enzyme (Akbarian et al., 2015; Qin and Hou. 2016; Mountzouris et al., 2020; Griela et al., 2021).

**Figure 1:**
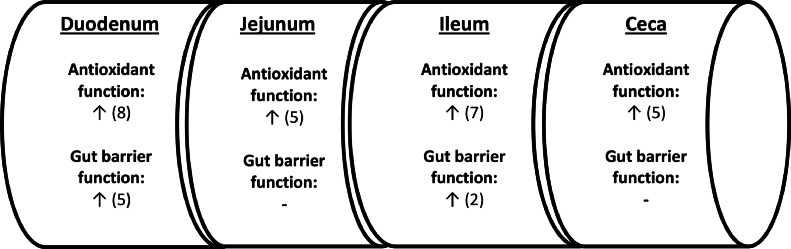
Summary of number and direction (↑↓) of changes shown for treatment M (blend of isoquinoline alkaloids, provided as Sangrovit Feed, Phytobiotics Futterzusatzstoffe GmbH, Germany) compared to treatment NC, regarding the antioxidant capacity and gut barrier function components analyzed for their relative gene expression along the 10d old broilers intestine.

In this work, the dietary supplementation of the IQs blend strongly modulated the antioxidant capacity at all intestinal segments (e.g., duodenum, jejunum, ileum, and ceca) as it was evidenced via the up-regulation of the majority of Nrf2 pathway genes studied. However, the duodenum was found to be the most responsive intestinal site to the supplementation of IQs blend, as 8 from the 11 Nrf2 pathway related genes assessed were significantly affected (Figure 1). These results might be attributed to the absorption and metabolism kinetics of isoquinoline alkaloids along the broiler intestine, and the notion that various phytobiotic bioactive compounds are considered to be mostly absorbed mainly at the proximal intestinal sites (Brenes and Roura. 2010).

In addition, in agreement with the results of the present study, up-regulation of the expression of *Nrf2, HMOX1, SOD1, SOD2,* and *GPX1* antioxidant genes in the broiler gut has been shown following dietary supplementation of isoquinoline alkaloids such as quaternary benzo[*c*]phenanthridine alkaloids (Liu et al., 2022). Beyond the direct antioxidant relevance of the results above, the general activation mode of Nrf2 pathway may also inhibit *NF-κΒ* activation, which in turn may down-regulate inflammation (Mountzouris et al., 2020; Kikusato et al., 2021a), but this remains to be specifically studied. Moreover, the upregulation of the expression levels of several Phase II antioxidant and cytoprotective enzymes, (e.g., CAT, SOD, GPX2, GPX7, GST, GSR, NQO1, HMOX1, PRDX1) highlights the protective role of IQs blend against oxidative threats in the intestinal environment of broilers at their early growth stage (Brouklogiannis et al., 2023).

The activation of the Nrf2 pathway upregulated the expression of many Phase II enzymes (e.g., SOD1, GPX2, GPX7, GSR, PRDX1, HMOX1), indicating a potential protective effect of PP against oxidative threat in the ovaries

In this work, the IQs blend enhanced the gene expression levels of the gut barrier components studied to a variable extend depending on the gut segment analyzed. In particular, the effects were noted primarily in the duodenum followed by the ileum as summarized in Figure 1. The impact of the IQs blend dietary addition on gut barrier integrity related genes, in combination with the results on antioxidant response, have shown that its bioactive components are probably absorbed and metabolized in the broiler upper intestinal level, where IQs blend effects are stronger regarding the investigated molecular biomarkers. Moreover, it has been reported that phytobiotic formulations based on thymol and carvacrol (Du et al., 2016), carvacrol, anethol, and limonen (Paraskeuas and Mountzouris. 2019a), menthol and anethole (Paraskeuas and Mountzouris. 2019b) and thymol, vanillin, and eugenol (Stefanello et al., 2020) could promote gut barrier via the expression of TJs. Moreover, *Glycyrrhiza glabra* (licorice) extract that is rich in flavonoids positively influenced gut barrier composition by elevating the expression levels of *MUC2* and *OCLN* (Ibrahim et al., 2020), whereas the flavonoid quercetin enhanced the stability of intestinal barrier via increasing relative expression of *OCLN* and *MUC2* at ileum (Dong et al., 2020). These effects could be linked to the bioactive compound composition in the supplemented phytobiotic formulations and to their dietary inclusion levels (Du et al., 2016; Paraskeuas and Mountzouris, 2019a; Stefanello et al., 2020). In this sense, IQs have been reported to promote the expression of tight junction proteins in broiler jejunum such as *ZO1* (Liu et al., 2022; Song et al., 2023) and *OCLN* (Song et al., 2023), *CLDN1* and *MUC2* (Khongthong et al., 2023).

Overall, the modulation of antioxidant capacity and the gut barrier by IQs blend dietary addition in the present study, could be considered concomitant with an enhanced protection potential. Interestingly, the latter protection potential for young broilers came at no additional cost for performance compared to the control treatment.

In conclusion, the dietary function of IQs blend was assessed with respect to the gut antioxidant capacity and gut barrier of young broilers at their starter growth period. The phytogenic blend used in the present study, which was characterized by components such as IQs, was shown to modulate the gut function of young broilers as they upregulated the majority of the antioxidant capacity and gut barrier components assessed. Although there were no performance benefits from IQs dietary addition, the findings demonstrate an enhanced potential for gut cytoprotection with no additional performance cost. The phytogenic blend merits further research not only with respect to a full production cycle but also with respect to various stressor challenges that could exacerbate oxidative and dysbiotic threats before further conclusions could be made.
